# An Electromagnetic Time-Reversal Imaging Algorithm for Moisture Detection in Polymer Foam in an Industrial Microwave Drying System

**DOI:** 10.3390/s21217409

**Published:** 2021-11-08

**Authors:** Adel Omrani, Rahul Yadav, Guido Link, Timo Lähivaara, Marko Vauhkonen, John Jelonnek

**Affiliations:** 1 Institute for Pulsed Power and Microwave Technology (IHM), Karlsruhe Institute of Technology (KIT), 76344 Eggenstein-Leopoldshafen, Germany; guido.link@kit.edu (G.L.); john.jelonnek@kit.edu (J.J.); 2 Department of Applied Physics, University of Eastern Finland, FI-70210 Kuopio, Finland; rahuly@uef.fi (R.Y.); timo.lahivaara@uef.fi (T.L.); marko.vauhkonen@uef.fi (M.V.); 3 Institute of Radio Frequency Engineering and Electronics (IHE), Karlsruhe Institute of Technology (KIT), 76131 Karlsruhe, Germany

**Keywords:** microwave drying, microwave tomography, time-reversal imaging, dyadic Green’s function, multilayered media

## Abstract

Microwave tomography (MWT) based control is a novel idea in industrial heating systems for drying polymer foam. In this work, an X-band MWT module is designed and developed using a fixed antenna array configuration and integrated with the HEPHAISTOS industrial heating system. A decomposition of the time-reversal operator (DORT) algorithm with a proper Green’s function of multilayered media is utilized to localize the moisture location. The derived Green’s function can be applied to the media with low or high contrast layers. It is shown that the time-reversal imaging (TRI) with the proposed Green’s function can be applied to the multilayered media with a moderately rough surface. Moreover, a single frequency TRI is proposed to decrease the measurement time. Numerical results for different moisture scenarios are presented to demonstrate the efficacy of the proposed method. The developed method is then tested on the experimental data for different moisture scenarios from our developed MWT experimental prototype. Image reconstruction results show promising capabilities of the TRI algorithm in estimating the moisture location in the polymer foam.

## 1. Introduction

Drying by microwaves has been widely used, especially in industry, for different applications and purposes. Microwaves can penetrate into the material and provide volumetric heating in contrast to the conventional drying procedure. In microwave drying applications, providing sufficient uniformity of heating distribution is an imperative task especially in industrial-scale production. Not meeting this condition can lead to hot-spot formation and thermal runaway [1]. These can decrease the efficiency of the drying system, threaten safety issues, and can lead to low-quality processing. In addition to the mentioned items, reducing the drying time and consumed energy and process intensification are also worthwhile for industry. It is well known that microwave heating is a nonlinear process which means that there is an increase in the dielectric loss factor with increasing temperature as the drying progresses leads to a rapid rise in the rate of change of temperature, causing a thermal runaway effect. This necessitates online intelligent control of the input power to prevent overheating. Thus, in most industrial heating systems temperature is used as the feedback parameter for online control [2]. Temperature output can be used to (i) switch the input power off at a set temperature, (ii) maintain the temperature at a set level for a specific period, or (iii) achieve a constant rate of heating. An excellent overview on the control strategies for the industrial microwave oven is provided in [3,4]. In [5], for a continuous oven type for batch processing, intelligent temperature control was demonstrated by controlling the power of distributed microwave power sources (magnetrons). However, for a nonuniform moisture distribution case in the samples, a temperature controlled process alone is not sufficient as the electromagnetic heating might cause some part of the samples to be over-dried and with progress in drying, catastrophic conditions can occur. Therefore, for precise control of the distributed magnetrons in this case [6], control requires in situ and noninvasive measurement of the unknown distribution of moisture inside the samples.

**Figure 1:**
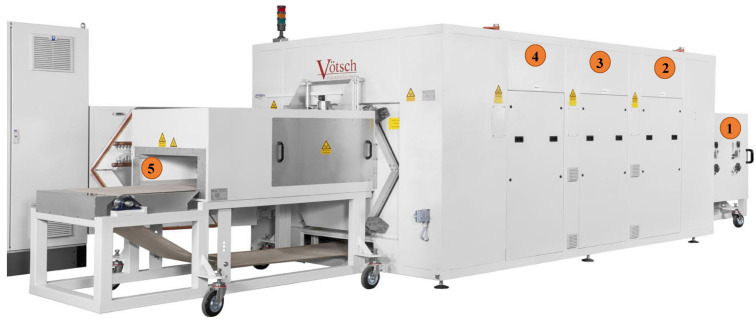
A side view of the HEPHAISTOS microwave oven system. Main modules of the oven are represented by number tags 1, 2, 3, 4, 5. Tag 1 and Tag 5 represent the entrance of the wet foam and exit doors for the dry foam on the conveyor belt, respectively. Tags 2, 3, 4 indicate the three modular heating systems which are built into the hexagonal cavity with high power microwave heating sources and control system block.

The microwave drying system which we are currently working with is named HEPHAISTOS (shown in Figure 1) from Weiss Technik Gmbh, Reiskirchen, Germany. This industrial microwave applicator has a patented hexagonal [7] cross-section design delivering high electromagnetic field homogeneity during the drying process at an operational frequency of 2.45 GHz. A conveyor belt, supported on a metal plate, is installed that enables a continuous drying process with a speed of 20 cm/min. Material processing, for instance, thermal curing of fiber composites, and drying of polymer foams (penetration depth is between about 2 m and 12 cm for moisture ranging from 0% to 100%), are some main target applications. For temperature controlled processes, infrared (IR) camera and fiber optic sensors are integrated with the HEPHAISTOS. The IR camera is limited to providing surface temperature and fiber optic sensors are not applicable in a running belt process. A microwave tomography (MWT) system is designed and integrated with the HEPHAISTOS to recover the volumetric information of the moisture location and its level during the drying process of the polymer foam. The power level and pulse duration of the magnetrons will be adjusted based on the input information from MWT. Some applications of the MWT in industry for inspection, monitoring purposes, quality control of the goods, and safety issues are listed in [8,9,10].

Designing and integrating an MWT system into an industrial microwave drying system is a challenging task. First, as mentioned earlier, the operational frequency of the HEPHAISTOS oven is 2.45 GHz, and it delivers a total of 36 kW EM power. Hence, the unwanted EM leakage power coming from the entrance aperture can take down the MWT system and it has to be blocked. In this regard, many types of antennas which are already employed for microwave imaging purposes cannot be used in this application, like corrugated tapered slot antennas [11], ultra wideband (UWB) tapered slot antennas [12], cavity backed aperture stacked patch antennas [13], and Vivaldi antennas [14], etc. Here, the X-band frequency range for the MWT is chosen, and WR-90 open-waveguide [15,16] is employed to act simultaneously as an antenna and microwave high pass filter. Being in the cut-off region of the WR90 waveguide for the X-band, the EM field decays with $e^{-\alpha z}$ (*z* is the direction of propagating) at 2.45 GHz where α is the attenuation constant, providing more than 100 dB isolation along with the waveguide antenna. Second, fast data acquisition is also essential to enable real-time monitoring and for process control. Hence, a limited number of antennas are chosen and arranged in a limited-aspect configuration. Note that here the requirement for matching media is not necessary due to the low contrast of the foam; even if any matching media allow improvement, their placement above/surrounding the foam in a continuous process will not be feasible. Third, for continuous or batch processing, a metal plate is installed in a conveyor belt system that should be taken into account in the forward model/physical formulation. Otherwise, image reconstruction may be erroneous. For image reconstruction which can support little data from a small number of antennas, neural networks [17,18,19] have shown promising capabilities that may estimate the moisture in real time (<1 s) [20,21] in comparison to an iteration based imaging algorithm. However, their performance can be plagued with changes in foam type, dimensions, and electrical properties; in that case the network requires retraining.

In this work, we use qualitative time-reversal imaging (TRI) for detecting the moisture location inside the polymer foam. Time-reversal imaging has been applied in numerous practical applications of ultrasonic [22,23,24,25] and electromagnetics. It is also used in medical applications such as diagnosis and treatment of breast cancer [26], and to keep track of kidney stones for lithotripsy processes [27]. Further, this method is employed in nondestructive testing, such as detection of defects in pipelines [28] and solids [29], and retrieving the permittivity of a hidden dielectric target in a cylindrical multilayered structure [30]. The high-resolution characteristic of TR facilitates the detection of the targets even under strong clutter conditions [31,32]. To apply the TRI, an exact or approximate dyadic Green’s function (DGF) of the medium is required [33]. In [34,35,36,37,38,39], only the transmission part of Green’s function was considered to locate the target in the multilayered media. However, it is not sufficient to take into account the presence of metal plates or reflecting surfaces below the foam. Therefore, we construct an approximate expression of the DGF by incorporating both the transmission and reflection parts of the multilayered media. The closed-form representation of the DGF is obtained by applying stationary phase approximation (SPA). Here, we would like to emphasise that in diffraction tomography based algorithms, e.g., UDT [38], MUDT [39], incorporating the reflected part will render the integral undefined as the closed form of the object function cannot be evaluated by applying SPA. Furthermore, a new single-frequency (SF) TRI-DORT is introduced based on the behavior of eigenvalues of the time-reversal operator (TRO) to foster high-speed data acquisition. Performance of the TRI and SF-TRI algorithms is tested with the numerical data considering the static condition case under scenarios without metal plate backing, with metal plate backing, and for nonideal conditions where the top surface of the foam is considered rough. Finally, the outlined methods are validated on the MWT data from our developed experimental prototype.

The paper is organized as follows: Section 2 provides an overview of the formulation for the TRI-DORT. Furthermore, an approximate expression for the DGF of the multilayered media is obtained using the SPA method. In Section 3, various numerical scenarios are investigated. Experimental results are investigated in Section 4, and finally Section 5 shows the discussion and concluding remarks.

## 2. Problem Formulation

**Figure 2:**
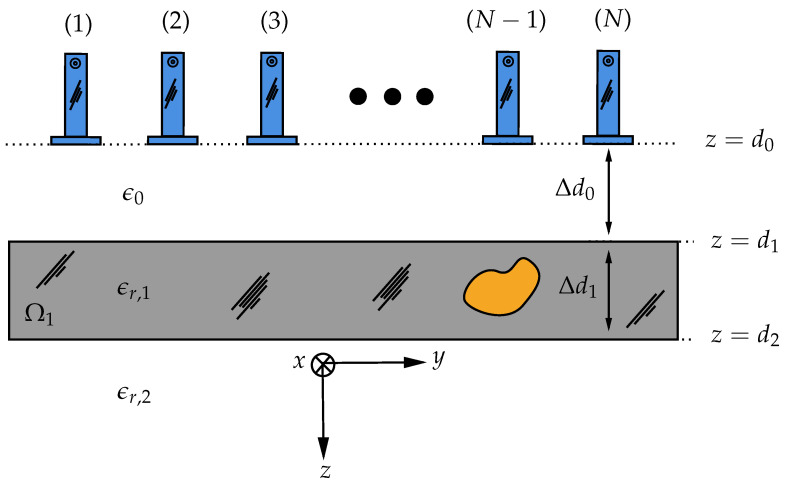
Multilayer media with the wet spot(s) in the second layer illuminated by the open waveguide antennas.

The imaging setup for a fixed cross-section (i.e., y×z) is shown in Figure 2 where layer 0 is the free space, layer 1 represents the polymer foam with thickness $\Delta d_{1}$, and layer 2 is either free space or a perfect electric conductor (PEC). The multistatic antenna array with *N* elements is fixed and the distance of the antennas to the top of the polymer foam is $\Delta d_{0}$, and the center to center distance between two adjacent antennas is *d*. In free space, the relative dielectric constant is denoted as $\epsilon_{0}$ whereas the relative dielectric constants of layer 1 and layer 2 are set to $\epsilon_{r,1}$ and $\epsilon_{r,2}$, respectively. In the next section, the multistatic data matrix (MDM) for the electric field will be built and an asymptotic expression for the Green’s function of the multilayer media will be obtained and used in TRI.

### 2.1. Scattering Model and Time-Reversal Imaging

Consider an active array of *N* transceivers from which an N×N MDM is constructed. Each element of the MDM matrix represents the received scattered field by the lth(l=1,2,…,N) antenna when the sth(s=1,2,…,N) antenna is in the transmitting mode due to the inhomogeneities in the region of interest (ROI) as follows [40,41,42]

(1) $$\begin{array}{c} {\vec{E}}_{\mathrm{sct}}^{\left(1\right)}\left({\vec{\rho }}_{r_{l}},{\vec{\rho }}_{t_{s}}\right)=i\omega \mu_{0}\int_{\Omega_{1}}{\overset{=}{G}}_{eb}^{\left(01\right)}\left({\vec{\rho }}_{r_{l}},{\vec{\rho }}^{\prime }\right)\;\cdot \;O\left({\vec{\rho }}^{\prime }\right){\vec{E}}_{\mathrm{tot}}^{\left(1\right)}\left({\vec{\rho }}^{\prime }\right)d{\vec{\rho }}^{\prime }. \end{array}$$

Here, ${\vec{E}}_{\mathrm{tot}}^{\left(1\right)}$ denotes the total electric field in layer 1 and ${\vec{E}}_{\mathrm{sct}}^{\left(1\right)}$ is the scattered field received by the lth antenna. $O\left(\rho^{\prime }\right)=-i\omega \epsilon_{0}\left(\epsilon_{\Omega_{1}}-\epsilon_{r,1}\right)$ is the object function where $\epsilon_{\Omega_{1}}$ denotes the target relative dielectric constants in the domain $\Omega_{1}$, and $\mu_{0}$ denotes free-space permeability. Here, time convention of $e^{-i\omega t}$ is assumed and suppressed where ω is the angular frequency. In (1), the vectors ${\vec{\rho }}_{r}$ and ${\vec{\rho }}_{t}$ represent source and observation points while ${\vec{\rho }}^{\prime }=\left(y^{\prime },z^{\prime }\right)$ is the location of the pixel point in the ROI. ${\overset{=}{G}}_{eb}^{\left(01\right)}$ is the background (multilayer media without any inhomogeneities) dyadic Green’s function (DGF). The superscript (01) denotes that the source point is located in layer 1 and the observation point is in layer 0. It should be noted that the analytical equations are written for the electric fields, however, in a real scenarios, the S-parameters will be measured. Hence, a de-embedding procedure will be applied later to relate the measured S-parameters to the electric field. The details of the employed procedure are represented in [36,37,43].

Under the assumption of excitation of the medium by a point source and using the Born approximation [41,44], the total electric field in layer 1 can be replaced by the background Green’s function of that layer. Consequently, a multistatic model for the received scattered field that describes each element of the MDM matrix can be written as

(2) $$\begin{array}{c} E_{\mathrm{sct}}^{\left(1\right)}\left({\vec{\rho }}_{r_{l}},{\vec{\rho }}_{t_{s}}\right)=i\omega \mu_{0}\int_{\Omega_{1}}G_{eb}^{xx}\left({\vec{\rho }}_{r_{l}},{\vec{\rho }}^{\prime }\right)O\left({\vec{\rho }}^{\prime }\right)G_{eb}^{xx}\left({\vec{\rho }}^{\prime },{\vec{\rho }}_{t_{s}}\right)d{\vec{\rho }}^{\prime }. \end{array}$$

In deriving (2) from (1) the symmetry property [42] of the DGF is used and, furthermore, it is assumed that both transmitting and receiving antennas are *x*-polarized and equivalent currents are predominantly *x*-polarized, so the xx term of the DGF is employed [34]. The MDM can be expressed in the compact form in the angular frequency domain ω as

(3) $$\begin{array}{c} K\left(\omega \right)=i\omega \mu_{0}\int_{\Omega_{1}}O\left({\vec{\rho }}^{\prime }\right)g_{b\rho_{l}}\left({\vec{\rho }}^{\prime },\omega \right)g_{b\rho_{s}}^{{}^{⊤}}\left({\vec{\rho }}^{\prime },\omega \right)d{\vec{\rho }}^{\prime }, \end{array}$$

where $g_{b\rho }={\left[G_{eb}^{xx}\left(\rho ,\rho_{1}\right),G_{eb}^{xx}\left(\rho ,\rho_{2}\right),...,G_{eb}^{xx}\left(\rho ,\rho_{N}\right)\right]}_{N\times 1}^{{}^{⊤}}$ is the frequency-domain steering vector of the xx components of the background DGF and ${\left(\;\cdot \;\right)}^{⊤}$ is the transpose operator. The decomposition of the time-reversal operator (DORT) can be applied to selectively focus on the inhomogeneities in the medium using singular value decomposition (SVD) of the MDM matrix [32]. The SVD of the matrix K(ω) is expressed in terms of the eigenvalues via the equation $K\left(\omega \right)=U\left(\omega \right)\Lambda \left(\omega \right)V^{†}\left(\omega \right)$, where Λ$\left(\mathrm{diag}(\Lambda )=[\lambda 1,\lambda 2,\ldots ,\lambda N]\right)$ is a real diagonal matrix consisting of eigenvalues, while *U*$\left(u_{l}\left(\omega \right),l=1,2,\ldots ,N\right)$ and *V*$\left(v_{l}\left(\omega \right),l=1,2,\ldots ,N\right)$ are the left and right matrices consisting of normalized eigenvectors [32] of time-reversal operator (TRO) formed as follows:

(4a) $$\begin{array}{cc} \lambda_{l}\left(\omega \right) & =\sigma_{l}\left(\omega \right){\left\|g_{b\rho_{l}}\left(\vec{\rho }\right)\right\|}^{2}, \end{array}$$

(4b) $$\begin{array}{cc} u_{l}\left(\omega \right) & =\frac{g_{b\rho_{l}}\left(\vec{\rho }\right)}{\|g_{b\rho_{l}}\left(\vec{\rho }\right)\|}, \end{array}$$

(4c) $$\begin{array}{cc} v_{l}\left(\omega \right) & =\frac{g_{b\rho_{l}}^{*}\left(\vec{\rho }\right)}{\|g_{b\rho_{l}}\left(\vec{\rho }\right)\|}. \end{array}$$

The number of nonzero significant eigenvalues (EVs) denotes the number of dominant inhomogeneities present in the background medium and the remaining eigenvalues form the noise subspace. For focusing on the pth scatterer, we employ $e_{p}\left(\omega \right)=\lambda_{p}\left(\omega \right)u_{p}\left(\omega \right)$ as a new excitation [32] for the transmitting antennas and calculate the propagated fields using the xx component of the background DGF. Here, $\lambda_{p}$ is the pth eigenvalue and $u_{p}$ is the pth left singular vector. As a result, the location of the pth scatterer is synthetically obtained by the following imaging function

(5) $$\begin{array}{c} D_{p}\left(\rho \right)=\int_{\omega }e_{p}^{T}\left(\omega \right)g_{b\rho }\left(\rho ,\omega \right)d\omega , \end{array}$$

where ρ is any arbitrary point in the ROI. In the next section, we obtain Green’s function of the multilayered media for image reconstruction using TRI-DORT.

### 2.2. Dyadic Green Function of Multilayered Media

Here, we obtain an asymptotic expression for the xx term of the DGF of the multilayered media. Previously, an asymptotic expression for the half-space was derived in [34] and used in TRI. However, this expression cannot be employed in the multilayered media with the high-contrast layer since the reflected wave will no longer be negligible, for example, with a PEC plate at the end of the layer. To address this problem, we obtain a more general closed-form expression for the Green’s function of the layered media. As mentioned earlier, we assume that an *x*-polarized line source residing in layer 0 is illuminating the media. In this case, the spectral representations of the Green’s function in the source layer and the first layer are given by [40]

(6) $$\begin{array}{cc} {\overset{=}{G}}^{\left(00\right)}\left({\vec{\rho }}_{r},{\vec{\rho }}_{t}\right) & =\int_{-\infty }^{+\infty }\frac{1}{k_{z0}}[T_{0}^{TM}\left(k_{y}\right)e^{-ik_{z0}\left(z-d_{0}\right)}\\ & +R_{0}^{TM}\left(k_{y}\right)e^{+ik_{z0}\left(z-d_{0}\right)}]\;\cdot \;e^{-ik_{zn}\left(z-d_{0}\right)}dk_{y}\;\;\;\;z>z^{\prime } \end{array}$$

(7) $$\begin{array}{cc} G_{eb}^{\left(n0\right)}\left(\vec{\rho },{\vec{\rho }}_{t}\right) & =\frac{1}{\pi }\int_{-\infty }^{+\infty }\frac{1}{k_{z0}}[T_{n}^{TM}\left(k_{y}\right)e^{-ik_{zn}\left(z-d_{0}\right)}\\ & \;\;\;\;+\;R_{n}^{TM}\left(k_{y}\right)e^{+ik_{zn}\left(z-d_{0}\right)}]\;\cdot \;\;e^{-ik_{y}\left(y-y_{t}\right)}dk_{y}. \end{array}$$

The details of the calculations are provided in Appendix A.

**Figure 3:**
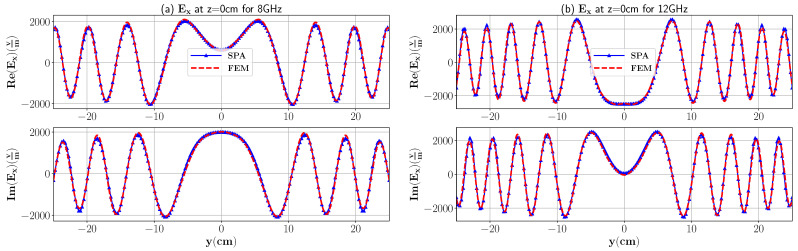
The real and imaginary part of the total electric field inside three-layer media with dimensions 30 cm × 8 cm and $\epsilon_{r,1}=1.16$ for the dielectric layer at 8 GHz and 12 GHz. The observation point is in the middle of the first layer, i.e., z=0 cm and −25 cm≤y≤25 cm. Layer 2 is free space.

In Figure 3, we have compared the electric field response at 8 GHz and 12 GHz from COMSOL multiphysics simulation software based on the finite element method (FEM) and our analytical formulations computed in MATLAB R2018b. For the COMSOL simulation, the imaging domain consists of foam with a domain size y∈[−25,25],z∈[−4,4] with relative dielectric constant $\epsilon_{r,1}=1.16-0.01i$ and placed in the background domain Ω consisting of air with $\epsilon_{0}=1-0i$. Open regions are truncated by using a perfectly matched layer (PML) of a circular shape with an inner radius and outer radius of 80 cm and 90 cm, respectively. The imaging medium is excited by a line current with an amplitude of 1 A and placed at (0 cm, 16 cm). The following parameters are set to define the triangular mesh in the model: maximum element size = 0.5 cm, minimum element size: $3.6\times 10^{-2}$ cm, maximum element growth rate = 1.1, curvature factor = 0.2. To simulate the electric field response, the RF module is employed with electromagnetic wave (emw) interface in the frequency domain. It is clear that the results of the FEM and numerical integration are in excellent agreement. Moreover, to better represent the differences between the analytical model and the FEM, the normalized root mean square (NRMS) error is calculated and the result is shown in Table 1.

## 3. TRI-DORT Simulation Results

**Figure 4:**
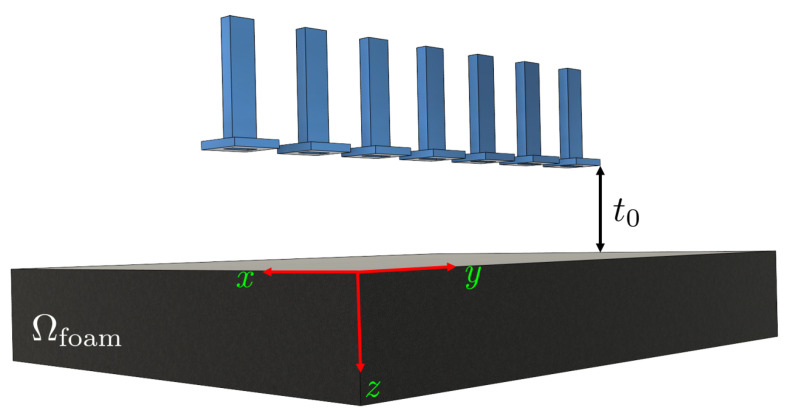
3-D MWT setup used in this study to generate synthetic measurement data.

In this section, TRI-DORT simulation results are demonstrated using the derived asymptotic Green’s function for different scenarios. To generate the synthetic data, the 3-D free-space MWT setup shown in Figure 4 is modeled in the commercial software CST Studio Suite. In the setup, foam of size $\Omega_{\mathrm{foam}}=\left[-25,\;25\right]\times \left[-15,\;15\right]\times \left[-4,\;4\right]$ cm is chosen. Seven WR90 open-ended waveguide antennas are placed with a center to center separation distance of 5 cm and located on top of the foam at a distance of $t_{0}=16\;\mathrm{cm}$. The radiation condition is satisfied by truncating the free space using a perfectly matched layer. The bottom of the foam is considered free space. However, to represent the metal plate below the conveyor belt, it is replaced by a perfect electric conductor (PEC) plate which resembles a high-contrast scenario. The time-domain solver of CST is chosen to simulate the EM fields. The 7×7 MDM matrix is extracted for the bandwidth of 4 GHz with the center frequency at 10 GHz (X-band range) and for $N_{f}=1001$ frequency points. It should be noted that the response of the antennas is removed from the measured S-parameter using a calibration proposed in [36,37]. In short, for the de-embedding process, two antennas are located in front of each other with a distance of *R* (here R=20 cm), and $S_{21}$ is stored. Then, using the following equation, the scattered electric fields from simulation or experiment scenarios are calculated

(8) $${\vec{E}}_{\mathrm{sct}}=i\omega \mu {}_{0}\frac{S_{{ls}_{tot}}-S_{{ls}_{free}}}{S_{21}}\frac{e^{-\mathrm{ikR}}}{4\pi R},$$

where $S_{ls_{tot}}$ and $S_{ls_{free}}$ are the scattered field with and without the target in the medium. In this study, the dielectric constant of the polymer foam is assumed to be known and is $\epsilon_{r,1}=1.16-0.01i$. Wet spots are given a spherical shape with the defined dielectric constant corresponding to the different moisture levels. The correlation between the dielectric constant and the wet-basis moisture levels is given in Table 2 [45]. The cavity-perturbation method as well as transmission line technique at 2.45 GHz are used for the measurement. It should be mentioned that the dielectric constant of the polymer foam is assumed to also be valid and constant in the X-band frequency range. For this characterization, the polymer foam density is $28\frac{\mathrm{kg}}{m^{3}}$. The TRI algorithm is implemented in MATLAB R2018b and its built-in SVD function is utilized to compute the eigenvalues of the MDM.

### 3.1. Low-Contrast Media

**Figure 5:**
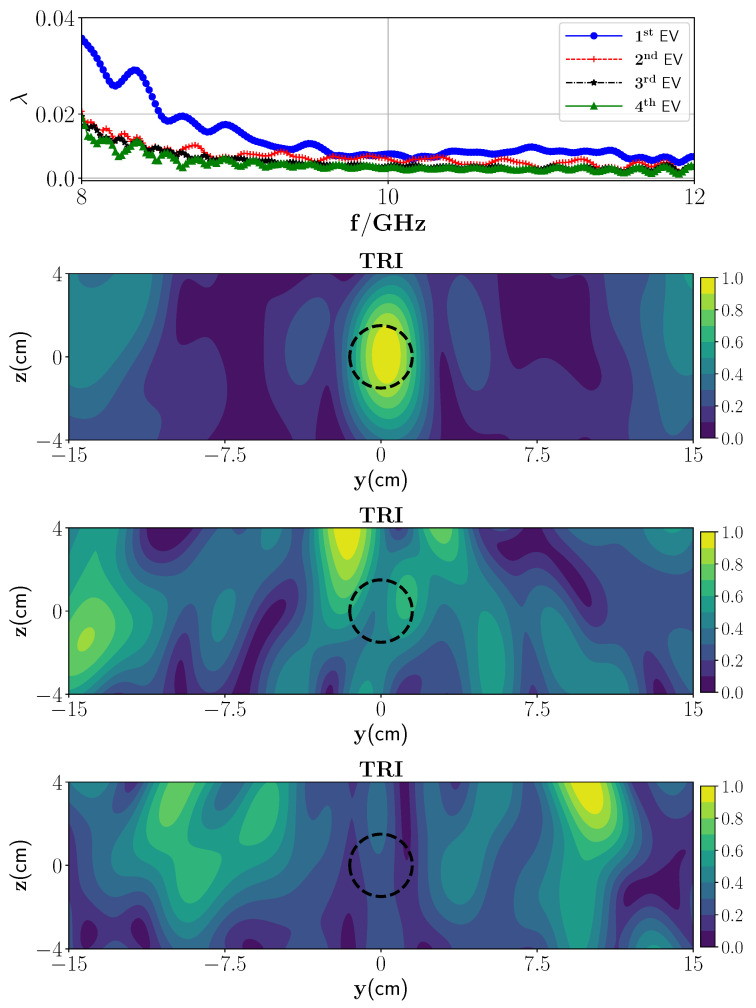
(**Top**) Magnitude of the first four eigenvalues versus the frequencies and (**second row**) reconstruction of one wet spot moisture case with TRI-DORT where the true location is marked by black dashed lines, (**third row**) reconstruction using third eigenvalue and its associated eigenvector, (**fourth row**) reconstruction using fourth eigenvalue and its associated eigenvector.

In the first scenario, the metal plate below the foam is not considered and foam is placed in infinite half-space. We consider one wet spot in the polymer foam with a radius of 1.5 cm located in the center of the polymer foam, i.e., (0 cm, 0 cm) in the yz plane with a 30% moisture level and surrounded by the dry part. Figure 5 (top) represents the behavior of the first four eigenvalues. According to Figure 5 (top), we expect one dominant wet spot in the polymer foam, and that the remaining eigenvalues construct the noise space (background). Figure 5 (second row) shows the reconstructed image associated with the first strong eigenvalue and employing the derived Green’s function. As can be seen, using TRI-DORT, the image domain is decomposed into the wet spot location and dry parts. Furthermore, reconstruction using nondominant (second and third) eigenvalues is also provided. As can be seen from Figure 5 (third and fourth row) and as mentioned earlier, these eigenvalues and associated eigenvectors represent the noise space.

**Figure 6:**
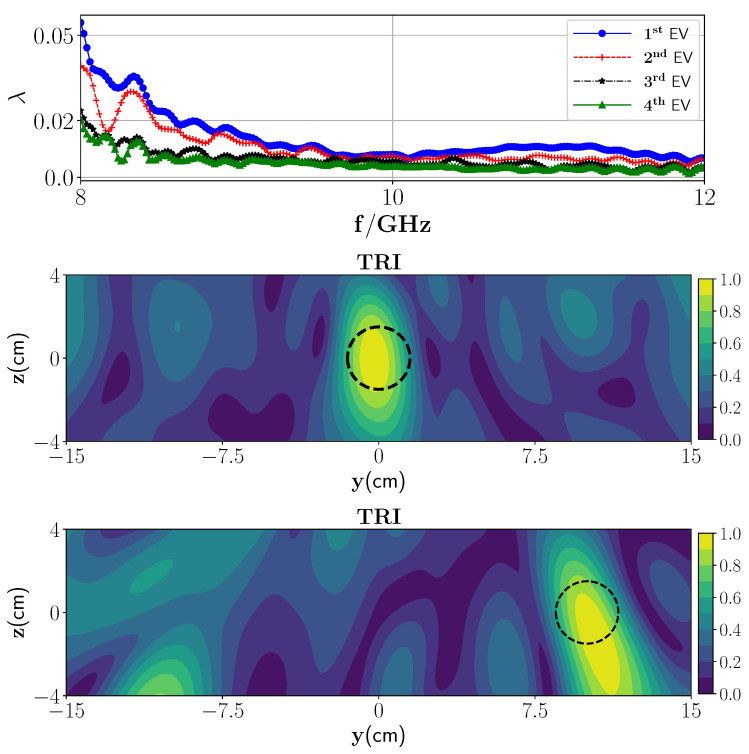
(**Top**) Magnitude of the first four eigenvalues versus the frequencies, (**middle**) reconstruction of the first wet spot moisture case with TRI using first dominant eigenvalue, and (**bottom**) reconstruction of the second wet spot moisture case with TRI-DORT using second dominant eigenvalue.

In the next scenario, two wet spots with a center to center distance of 10 cm and the same moisture level are considered in the polymer foam. The operation frequency of the HEPHAISTOS oven is 2.45 GHz, hence the range of the drying heating pattern is around $\frac{\lambda }{2}\approx 6\;\mathrm{cm}$. Resolving wet spots in this range is critical for proper and effective power conducting. By applying the SVD to the stored scattering matrix in different frequencies, the eigenvalues can be plotted. Figure 6 (top) represents the behavior of the first four eigenvalues. As can be seen, two dominant eigenvalues can be observed and the remained eigenvalues belong to the null space. The corresponding TRI-DORT images associated with the first and second eigenvalues are plotted in Figure 6 (second and third row). The imaging domain is decomposed into the individual wet spots and the correct locations are obtained and selectively focused.

### 3.2. High-Contrast Media

**Figure 7:**
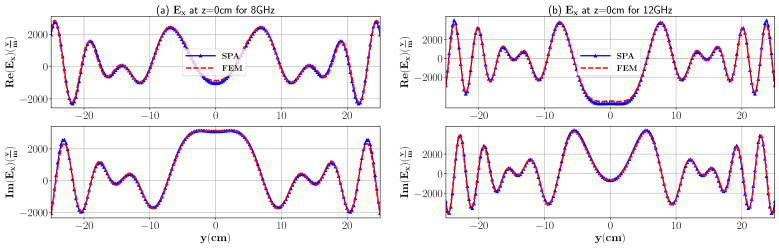
The real and imaginary part of the total electric field inside three-layer media with dimensions 30 cm × 8 cm and $\epsilon_{r,1}=1.16$ for the dielectric layer at 8 GHz and 12 GHz. Layer 2 is PEC.

In this case, a metal plate (PEC) at the bottom of the polymer foam is considered, i.e., the third layer shown in Figure 2. Due to the introduction of the PEC, a high-contrast dielectric constant variation between the different layers exists. In this scenario, the reflected wave from the interface would not be negligible and should be considered in the formulation. As mentioned earlier, Green’s function is composed of two parts that are transmission and reflection. Since the contrast between the layers is no longer negligible, both transmitted and reflected components of the EM field are considered in modeling of Green’s function, i.e., $G^{\left(10\right)}=G_{T}^{\left(10\right)}+G_{R}^{\left(10\right)}$. Figure 7 represents the comparison between the analytical model of Green’s function and FEM. The figures are plotted in the same lines as Figure 3. Moreover, to better represent the differences between the analytical model and FEM, the NRMS error is calculated and the result is shown in Table 3.

**Figure 8:**
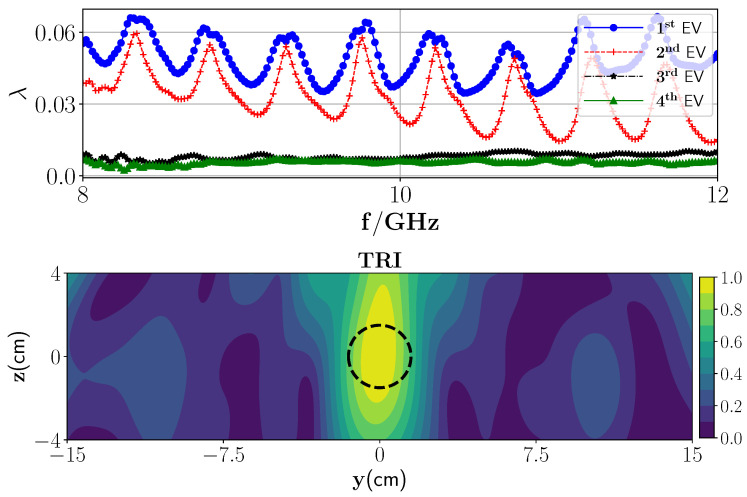

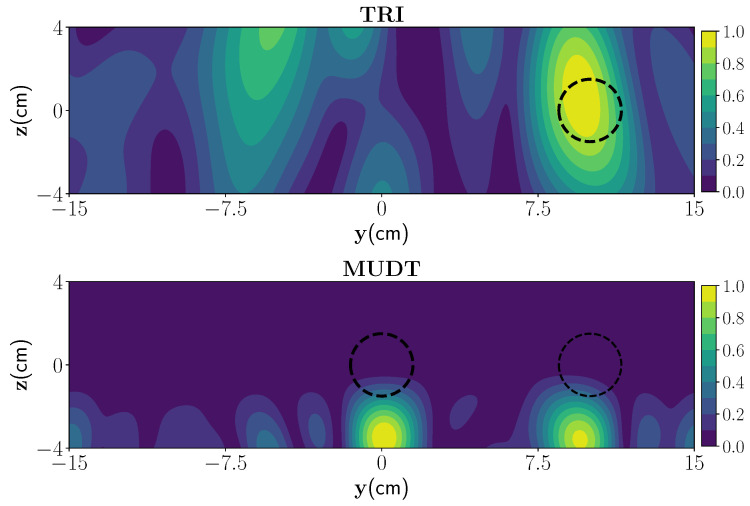
(**Top**) Magnitude of the first four eigenvalues versus the frequencies, (**second row**) reconstruction of the first wet spot moisture case with TRI-DORT using first dominant eigenvalue and (**third row**) reconstruction of the second wet spot moisture case with TRI-DORT using second dominant eigenvalue when the layer 2 is a PEC plate, (**fourth row**) reconstruction using MUDT imaging algorithm.

We consider the presence of the two wet spots inside the foam with the same specification as in the previous case. In addition, 3% Gaussian noise with zero mean is added to the data in this case to perform a numerical experiment. First and second dominant eigenvalues are employed to focus on the first and second targets, respectively. Reconstruction images with updated $G^{\left(10\right)}$ are depicted in Figure 8. The reconstructed images show that the two wet spots are properly detected. Upon comparison of the TRI reconstruction in Figure 6 (without PEC) and Figure 8 (with PEC), it can be seen that the presence of a PEC plate may give a better reconstruction than that with no PEC layer. This is due to the reflections from the PEC surface which increase the spatial resolution by providing more views of the targets [46,47].

Furthermore, we have compared the TRI method with a multistatic uniform diffraction tomography (MUDT) algorithm in which, as mentioned earlier, only the transmission part is incorporated. Reconstruction with MUDT is shown in Figure 8 (fourth row) which clearly depicts the erroneous target detection in this case.

### 3.3. Single Frequency TRI

In practical applications, fast data acquisition is a critical task. By decreasing the number of frequencies, the measurement time can be decreased. Single frequency TR multiple signal classification (SF-TRI-MUSIC) is proposed in [34,48] to increase the resolution of the reconstructed image after reconstructing using ultra-wideband (UWB) TRI-MUSIC. Here, we propose an SF-TRI-DORT by observing the behavior of the eigenvalues. The reconstructed image is formed using different orthogonal eigenvectors and corresponding eigenvalues [49]. The orthogonality of eigenvectors in the frequency domain indicates carrying independent information by each eigenvector. Hence, it provides a linear summation as represented in (9). In the SF-TRI, this linear summation is approximated with one eigenvector and the associated eigenvalue, i.e., $D_{p,SF}\approx D_{p}\delta \left(\omega -\omega_{SF}\right)$. To choose this frequency, we look at the dominant eigenvalues and compare them with those that belong to the null space. Bigger differences mean the target domain is more distinguishable from null space. The eigenvector associated with this eigenvalue $\left(v_{p,SF}\right)$ forms a new excitation for calculating the $D_{p,SF}$. So, instead of conventional TRI-DORT, a single frequency in this range is chosen for the image reconstruction

(9) $$\begin{array}{c} D_{p,SF}\left(\rho \right)=\lambda_{SF}v_{p,SF}g_{b\rho }\left(\rho ,\omega_{SF}\right) \end{array}$$

**Figure 9:**
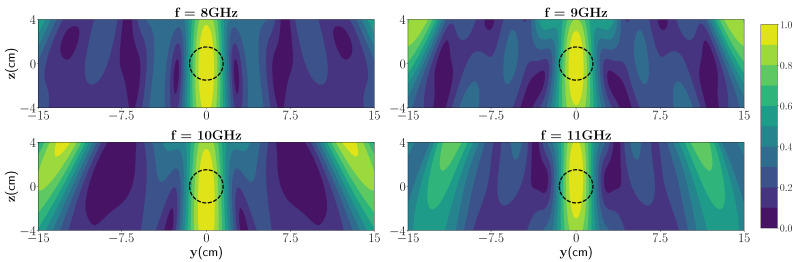
Reconstruction using SF-TRI-DORT at four frequencies.

From Figure 5 (top), it can be seen that for the low frequencies the difference between the dominant eigenvalue and those belonging to the null space is higher than at higher frequencies. The differences of the 1st eigenvalue and 2nd eigenvalue at 4 different frequencies are as follows: $\Delta f_{8\mathrm{GHz}}\times 10^{3}=6.7$, $\Delta f_{9\mathrm{GHz}}\times 10^{3}=2.5$, $\Delta f_{10\mathrm{GHz}}\times 10^{3}=2.0$, $\Delta f_{10\mathrm{GHz}}\times 10^{3}=2.7$, as shown in Table 4. So, we expect less shadow-images and higher resolution at 8 GHz than at other frequencies. Figure 9 shows the reconstruction using SF-TRI-DORT for one single target in the media in four different frequencies. As can be seen, the location of the wet spot is visible in the domain. However, the spatial resolution was slightly decreased by using a single frequency approach.

It can be perceived from Figure 9, since the eigenvectors are orthogonal in different frequencies, by decreasing the number of frequencies, less information from the media under investigation is available, which leads to decrease in spatial resolution compared to the multiple-frequencies reconstructed image. Also, the presence of multiple wet spots can plague the performance of the TRI algorithm for which the best case strategy is to use the multi-frequency TRI algorithm. To reduce the data acquisition time (DAQ) time for measurements of multiple frequencies, a specific band in the X-band can also be targeted. A pragmatic study in this direction is given in [50].

### 3.4. Moderately Rough Surface

Despite the previous scenarios, here we consider the foam with a moderately rough surface. This scenario is undertaken considering pre-preparation drying process defects. In order to investigate the performance of the TRI when the surface contains some roughness, a polymer foam with a randomly rough surface on the top interface is considered and modeled [51] as

(10) $$z\left(y\right)=\sum_{m=-M}^{M}m^{-\beta }G_{m}\cos\left(2\pi my+U_{m}\right).$$

Here, *m* is the integer number representing the spatial frequency and β denotes the spectral exponent, $G_{m}$ is sampled from the Gaussian distribution as $G_{m}∼N\left(\mu ,\sigma \right)$ with mean μ and standard deviation σ, and $U_{m}∼U\left(0,2\pi \right)$ is sampled from the uniform distribution as $U_{m}∼U\left(0,2\pi \right)$. Under the assumption of a moderately rough surface, the Green’s function can be replaced by the planar surface one. So, the asymptotic fields inside the polymer foam with the rough surface for the first interface are estimated using the SPA under the σ=0 and β=0.

**Figure 10:**
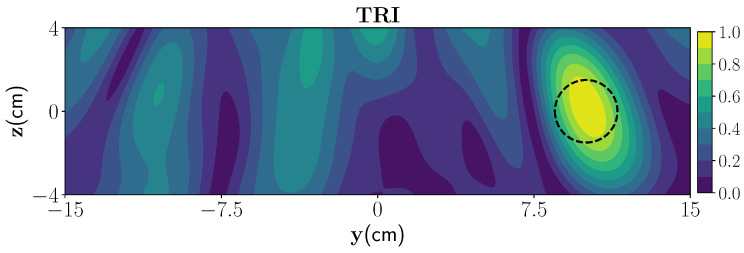
Reconstruction of one wet spot using TRI-DORT for moderate random rough surface.

To investigate the performance of the TRI, a wet spot with 30% moisture (1.69−0.1i) with the radius 1 cm at the position (10 cm, 0 cm) is considered inside the foam and a moderately rough surface with the following parameters: μ=0, σ=0.2, and β=0.8. Figure 10 shows the TRI constructed image. The location of the wet spot is visible but, compared to the polymer foam with the smooth surface, the shadow images are more prominent. It can be seen that, when increasing the roughness of the top interface, the electromagnetic fields inside the layer cannot be fully estimated by the current asymptotic expression (since the Green’s function is analytically calculated for the smooth multilayered media, and it cannot estimate the multiple scattering between the rough surface and bottom interface), and it leads to more strong shadow images in the reconstruction.

## 4. Experimental Results

**Figure 11:**
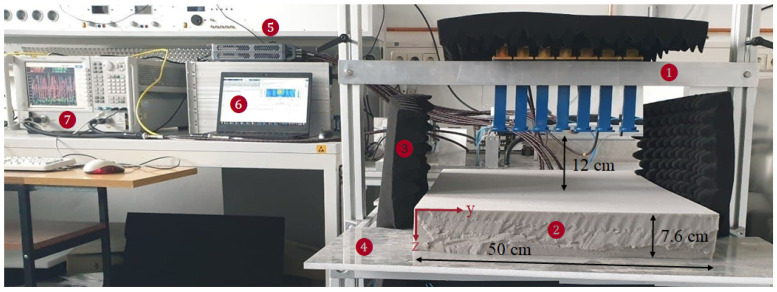
Prototype of MWT sensor array used in this study to generate measurement data. The MWT sensor configuration consists of 7 X-band open-ended waveguide antennas, indicated by Tag 1. The polymer foam is shown by Tag 2 and surrounded by absorbers as shown by Tag 3. The flexible PEC plate is shown by Tag 4. To acquire the measurement the solid switch, PC and VNA are used as shown by Tags 5, 6, and 7, respectively. This system is developed at KIT, Germany and will be integrated with the HEPHAISTOS technology in the final stage.

**Figure 12:**
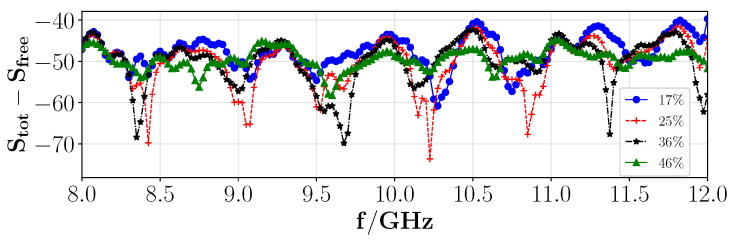
Experimental scattering response (in dB) in X-band of antenna 4 (middle antenna) for different moisture contents in the spherical wet spot of radius 1.5 cm.

This section is devoted to investigating the experimental TRI of polymer foam. As shown in Figure 11, the integrated MWT setup consists of 7 WR90 open-ended waveguide antennas (VSWR 1.03:1) connected (with the phase stable cables with phase stability $3^{{}^{\circ}}$ at maximum frequency) to a Agilent N5224A vector network analyzer (VNA) with a P9164C 2×16 USB solid state switch matrix with power level of 5 dBm, and IF bandwidth 500 Hz. It should be noted that a waveguide calibration is performed to extract the response of the medium from the stored scattering matrix. The data are acquired from 8 GHz to 12 GHz with a frequency step of 5 MHz. Communication between the VNA, switch, and the controlling computer is accomplished through the Ethernet cable. The data acquisition process is entirely automated using MATLAB R2018b. The multistatic antenna array is located in semi-infinite free space from −15 cm to 15 cm along the *y*-axis and the distance of the top antenna to the top of the polymer foam is 12 cm, and the center to center distance between two adjacent antennas is 5 cm. The setup is surrounded with the microwave absorber to increase the signal to noise ratio. The return loss of antenna 4 (middle antenna) for different moisture contents in the spherical we spot of radius 1.5 cm is shown in Figure 12 which clearly indicates the sensitivity of the measured data with respect to the change in moisture content.

**Figure 13:**
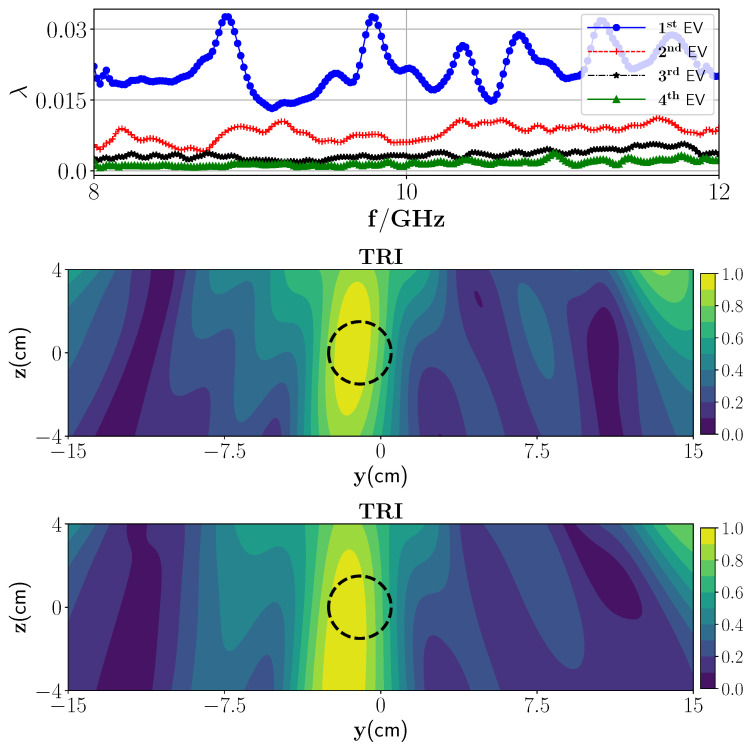
(**Top**) Magnitude of the first four eigenvalues versus the frequencies,(**middle**) TRI-DORT reconstruction image of one PTFE Teflon, and (**bottom**) SF-TRI-DORT at 8.07 GHz.

In the first experiment, as a low-contrast layered medium, one PTFE Teflon sphere (radius 1.5 cm) is placed inside the foam with the center position of (0 cm, −0.7 cm, 0 cm). In this case, we employ only the transmission part of the Green’s function ($G^{\left(10\right)}\approx G_{T}^{\left(10\right)}$) for image reconstruction. Furthermore, since the measurement response from the background media is known, we can suppress it from the total response using a background subtraction procedure. In essence, the detection problem is studied here in terms of residual measurement which is obtained by subtracting the measurement response from the foam with wet spots by the measurement response from the dry foam. Later, an antenna de-embedding is performed to convert the received S-parameters to the electric field. As can be seen from depicted eigenvalues in Figure 13 (top), there is one dominant eigenvalue, and the reconstructed image associated with this is shown in Figure 13 (middle). As can be observed from this figure, the location of the target can be reconstructed. To apply the proposed SF-TRI-DORT, the $f_{SF}$ is chosen based on the maximum difference between the first dominant eigenvalue and those belonging to the null space, which is here $f_{SF}=8.07$ GHz. Figure 13 (bottom) demonstrates the SF-TRI-DORT. The position of the target is truly obtained, however, the spatial resolution is decreased.

**Figure 14:**
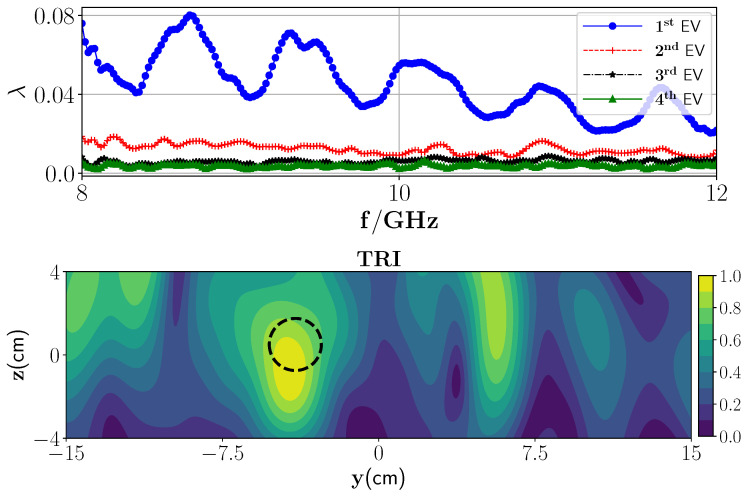
(**Top**) Magnitude of the first four eigenvalues versus the frequencies and (**bottom**) TRI-DORT reconstruction image of one wet spot when the second layer is PEC plate.

In the second experiment, we insert a PEC plate under polymer foam, as a high-contrast layered medium. A wet spot is inserted in the polymer foam. To create the wet spot moisture target, a spherical foam of diameter 2.5 ± 0.1 cm and with a 36% wet basis moisture level ($\epsilon_{r}\approx 1.87-0.12i$) is chosen. An approximate location of the target inside the foam is centered at (−3.5 cm, 1.2 cm, 0 cm). In this case, we employ the transmission and the reflection parts of the Green’s function ($G^{\left(10\right)}=G_{T}^{\left(10\right)}+G_{R}^{\left(10\right)}$) for image reconstruction. We repeat the same steps as in the previous experiment to extract the MDM matrix from the measurement data. The eigenvalue behavior of the MDM matrix is shown in Figure 14 (top) from which it can be observed that there is one dominant eigenvalue that indicates the presence of one wet spot in the media. The reconstructed TRI-DORT image associated with the first dominant eigenvalue is shown in Figure 14 which shows that the estimated position of the target is correctly obtained with good spatial resolution.

## 5. Conclusions and Discussion

In this paper, a time-reversal based imaging algorithm was proposed for the MWT application in the industrial microwave drying process to achieve intelligent process control. For testing and validating the proposed algorithm, we have focused first on a specific static case where it is applied to obtain the location of moisture in polymer foam. To formulate the problem, a closed-form Green’s function of the multilayered media was obtained using the SPA method and used in the TRI function. The developed TRI was applied to both low- and high-contrast media specifically when a low-dielectric layer is situated above a PEC layer. Additionally, the medium with a moderately rough surface was investigated as a nonideal condition. Furthermore, a single frequency SF-TRI was proposed to enable high-speed data acquisition from a limited number of antennas; a challenge in many industrial applications of MWT. The frequency for SF-TRI operation is chosen by selecting the frequency where the maximum difference between the dominant eigenvalue and those belonging to the null space exists. The proposed TRI and SF-TRI approach was demonstrated with numerical studies and tested on our developed experimental MWT prototype. The results were obtained in less than a second and show the efficiency of the proposed TRI and SF-TRI algorithm in accurately locating the considered targets under low-contrast and high-contrast medium conditions. However, a slight decrease in spatial resolution was noticeable in the SF-TRI method in comparison to the TRI.

Our future work concerns (i) integrating the MWT block with the microwave heating system and testing the developed TRI and SF-TRI method for foam moving on the conveyor belt. To give an idea of the dynamic measurement, consider foam with approximate width = 50 cm, height = 7 cm, and length = 100 cm. Therefore, taking into account the foam size and conveyor belt speed of 20 cm/min, we expect to reconstruct the moisture information in a length of 16 cm in 50 s, i.e., a total processing time which includes the measurement, data transfer, and image reconstruction. With this sampling rate, crucial information on the moisture content will be lost. Therefore, instead of full X-band, the measurements can be targeted for a specific band as a way to bring down the total processing time to around 11 s. Considering the heating pattern inside the oven cavity (between λ/4 to λ/2 at an operational frequency of 2.45 GHz of the oven), this may be sufficient for process control; (ii) retrieving the moisture level of the detected wet spots [52,53]; (iii) testing the process control performance with the MWT input; and (iv) in the final stages, possibly translating from the level of prototype design to commercial design.

## Figures and Tables

**Table 1:** NRMS error values for compared analytical model and FEM in Figure 3.

	Re(Ex),8GHz	Im(Ex),8GHz	Re(Ex),12GHz	Im(Ex),12GHz
NRMS %	1.76	2.06	2.11	2.74

**Table 2:** Permittivity of the foam with different moisture contents *M* on wet basis.

*M*%	0	30	36
$\epsilon_{r}$	1.16−0.01i	1.69−0.1i	1.81−0.16i

**Table 3:** NRMS value for compared analytical model and FEM in Figure 7.

	Re(Ex),8GHz	Im(Ex),8GHz	Re(Ex),12GHz	Im(Ex),12GHz
NRMS %	2.42	1.89	1.94	2.15

**Table 4:** The difference between the first dominant eigenvalue and those belonging to the null space at four different frequencies.

*f* (GHz)	8	9	10	11
$\Delta \lambda \times 10^{3}$	6.6654	2.029	2.4659	2.694

## Data Availability

The data presented in this study are available on request from the corresponding authors.

## Appendix A

The relation between transmission and reflection coefficients of the Green’s function in layer *n* in the TM${}_{x}$ case can be expressed as

(A1) $$\left[\begin{array}{c} R_{n+1}^{TM}e^{+ik_{z(n+1)}d_{\left(n+1\right)}}\\ T_{n+1}^{TM}e^{-ik_{z(n+1)}d_{\left(n+1\right)}} \end{array}\right]={\overset{=}{V}}_{n(n+1)}^{TM}\left[\begin{array}{c} R_{n}^{TM}e^{+ik_{zn}d_{n}}\\ R_{n}^{TM}e^{-ik_{zn}d_{n}} \end{array}\right]$$

where

(A2) $${\overset{=}{V}}_{n(n+1)}^{TM}=\frac{1}{2}\left(1+\frac{k_{zn}}{k_{z(n+1)}}\right)\times \left[\begin{array}{cc} e^{+ik_{z(n+1)}\Delta d_{n}} & -R_{n(n+1)}^{TM}e^{+ik_{z(n+1)}\Delta d_{n}}\\ -R_{n(n+1)}^{TM}e^{-ik_{z(n+1)}\Delta d_{n}} & e^{-ik_{z(n+1)}\Delta d_{n}} \end{array}\right]$$

$R_{n(n+1)}^{TM}$ is the Fresnel reflection coefficient for the interface between layers *n* and n+1 and is given by

(A3) $$R_{n(n+1)}^{\mathrm{TM}}=\frac{k_{zn}-k_{z(n+1)}}{k_{zn}+k_{z(n+1)}}.$$

$T_{n(n+1)}^{\mathrm{TM}}$ is the Fresnel transmission coefficient for the interface between layers *n* and n+1 and is given by

(A4) $$T_{n(n+1)}^{\mathrm{TM}}=\frac{2k_{zn}}{k_{zn}+k_{z(n+1)}}.$$

By considering only the first-order reflection and transmission coefficient inside each layer and after some straightforward calculations, an expression of the Green’s function with the source point in the 0th layer and observation point in layer *n*th can be derived as follows

(A5) $$\begin{array}{cc} G^{\left(n0\right)}\left({\vec{\rho }}_{r},{\vec{\rho }}_{t}\right) & =\frac{1}{\pi }\int_{-\infty }^{+\infty }\frac{1}{k_{z0}}\times [\prod_{q=0}^{n-1}T_{q(q+1)}^{\mathrm{TM}}e^{-i[k_{zn}\left(z-d_{0}-\sum_{q=0}^{n-1}\Delta d_{q}\right)+\sum_{q=0}^{n-1}k_{zq}\Delta d_{q}]}\\ & +\prod_{q=0}^{n-1}R_{q(q+1)}^{\mathrm{TM}}T_{q(q+1)}^{\mathrm{TM}}e^{+i[k_{zn}\left(z-d_{0}-\sum_{q=0}^{n-1}\Delta d_{q}\right)+\sum_{q=0}^{n-1}k_{zq}\Delta d_{q}]}]e^{-ik_{y}\left(y-y_{t}\right)}dk_{y}. \end{array}$$

The Green’s function in the above equation is composed of two parts and it can be written as a summation of the transmission and reflection terms, i.e., $G^{\left(n0\right)}=G_{T}^{\left(n0\right)}+G_{R}^{\left(n0\right)}$. In the following, we address each part separately and obtain a closed form for the Green’s function of the multilayer media. The stationary phase approximation estimates a solution for the oscillating integral as follows [54]

(A6) $$\begin{array}{cc} F(\kappa ) & =\int_{-\infty }^{+\infty }f\left(h\right)e^{i\kappa \phi (h)}dh\\ & \approx {\left[\frac{2\pi }{\kappa |\phi^{\prime \prime }\left(h_{0}\right)|}\right]}^{\frac{1}{2}}f\left(h_{0}\right)e^{i[\kappa \phi \left(h_{0}\right)-\frac{\beta }{2}+\frac{\pi }{4}]}, \end{array}$$

(A7) $$\begin{array}{c} \phi^{\prime \prime }\left(h_{0}\right)=\left[\phi^{\prime \prime }\left(h_{0}\right)\right]e^{i\beta }\;\;\phi^{\prime }\left(h_{0}\right)=0, \end{array}$$

where κ is real and large, *f* and ϕ are real and infinitely differentiable, so an infinite asymptotic expansion can be obtained via the integration-by-parts method [55]. Additionally, $h_{0}$ is the point where $\phi^{\prime }=0$, known as a stationary point. So, the first term of (A5) reduces to a simple summation given as

(A8) $$\begin{array}{c} G_{T}^{\left(n0\right)}=\frac{1}{\pi }\left(\prod_{q=0}^{n-1}T_{q(q+1)}^{\mathrm{TM}}\left(k_{y,sp_{1}}\right)\right){\left[\frac{2\pi }{|\Phi_{T}^{\prime \prime }\left(k_{y,sp_{1}}\right)|}\right]}^{\frac{1}{2}}\\ \;\;\;\;\;\;\times e^{-i[k_{y,sp_{1}}\left(y-y_{t}\right)+{\tilde{k}}_{zn}\left(z-d_{0}-\sum_{q=0}^{n-1}\Delta d_{q}\right)+\sum_{q=0}^{n-1}{\tilde{k}}_{zq}\Delta d_{q}]}e^{-i\left(\frac{\beta }{2}-\frac{\pi }{4}\right)} \end{array}$$

where

(A9) $$\Phi_{T}^{\prime \prime }\left(k_{y,sp_{1}}\right)=-\frac{k_{n}^{2}}{k_{zn}^{3}}\left(z-d_{0}-\sum_{q=0}^{n-1}\Delta d_{q}\right)+\sum_{q=0}^{n-1}\frac{k_{q}^{2}}{k_{zq}^{3}}\Delta d_{q}$$

(A10) $$\Phi_{T}^{\prime \prime }\left(k_{y,sp_{1}}\right)=\left|\Phi_{T}^{\prime \prime }\left(k_{y,sp_{1}}\right)\right|e^{-i\beta },$$

where $k_{y,sp1}$ is the stationary point and is the solution of the following equation

(A11) $$k_{y,sp_{1}}=\frac{y-y_{t}}{\sum_{q=0}^{n-1}\frac{\Delta d_{q}}{k_{zq}}+\frac{1}{k_{zn}}\left(z-d_{0}-\sum_{q=0}^{n-1}\Delta d_{q}\right)}$$

(A12) $${\tilde{k}}_{zn}=\sqrt{k_{n}^{2}-k_{y,sp1}^{2}}.$$

Analogous to the transmission term, a closed form for the reflection term can also be derived after some straightforward calculations, leading to

(A13) $$\begin{array}{c} G_{R}^{\left(n0\right)}=\frac{1}{\pi }\left(\prod_{q=0}^{n-1}T_{q(q+1)}^{\mathrm{TM}}\left(k_{y,sp_{2}}\right)R_{q(q+1)}^{\mathrm{TM}}\left(k_{y,sp_{2}}\right)\right){\left[\frac{2\pi }{|\Phi_{T}^{\prime \prime }\left(k_{y,sp_{2}}\right)|}\right]}^{\frac{1}{2}}\\ \;\;\;\;\;\times e^{-i[k_{y,sp_{2}}\left(y-y_{t}\right)+{\tilde{k}}_{zn}\left(z-d_{0}-\sum_{q=0}^{n-1}\Delta d_{q}\right)-\sum_{q=0}^{n-1}{\tilde{k}}_{zq}\Delta d_{q}]}e^{-i\left(\frac{\beta }{2}-\frac{\pi }{4}\right)} \end{array}$$

where

(A14) $$\Phi_{T}^{\prime \prime }\left(k_{y,sp_{2}}\right)=-\frac{k_{n}^{2}}{k_{zn}^{3}}\left(z-d_{0}-\sum_{q=0}^{n-1}\Delta d_{q}\right)-\sum_{q=0}^{n-1}\frac{k_{q}^{2}}{k_{zq}^{3}}\Delta d_{q}$$

where $k_{y,sp2}$ is the stationary point and is the solution of the following equation:

(A15) $$k_{y,sp_{2}}=\frac{y-y_{t}}{\sum_{q=0}^{n}\frac{\Delta d_{q}}{k_{zq}}-\frac{1}{k_{zn}}\left(z-d_{0}-\sum_{q=0}^{n-1}\Delta d_{q}\right)}$$
